# Genetic profiling of patients with adenoid cystic carcinoma of the Bartholin’s glands reveals potential new routes for targeted therapies: a case report

**DOI:** 10.1186/s13000-020-00976-2

**Published:** 2020-05-28

**Authors:** Kohei Nakamura, Eriko Aimono, Shigeki Tanishima, Hidetaka Nomura, Mitsuho Imai, Hideyuki Hayashi, Hiroshi Nishihara

**Affiliations:** 1 Genomics Unit, Keio Cancer Center, Keio University School of Medicine, 35 Shinanomachi, Shinjukuku, Tokyo, 160-8582 Japan; 2 Department of Obstetrics and Gynecology, Kumagaya General Hospital, Saitama, 360-8657 Japan; 3 Department of Biomedical Informatics, Kansai Division, Mitsubishi Space Software Co., Ltd, Tokyo, Japan; 4 Department of Gynecology, The Cancer Institute Hospital of Japanese Foundation for Cancer Research, Ariake 3-8-31, Tokyo, 135-8550 Japan

**Keywords:** Bartholin gland carcinoma, Adenoid cystic carcinoma, Genome sequencing, Precision medicine

## Abstract

**Background:**

Bartholin gland carcinomas (BGCs) are rare tumor types, for which no molecular analyses including genomic sequencing have been reported to date. Adenoid cystic carcinomas (ACCs) of the Bartholin’s glands are an atypical histological type of BGC, and currently nothing is known regarding their genetic profiles or similarity to ACC carcinogenesis in other organs including the salivary glands, thereby limiting possible therapeutic options using precision medicine.

**Case presentation:**

We used targeted gene sequencing to analyze the occurrence of 160 cancer-related genes in two patients with BG-ACC. *KRAS* and *KDM6A* mutations were detected in tumor samples collected from each patient. No KRAS mutations have been previously reported in salivary gland ACCs, indicating that the carcinogenesis of BG-ACC differs from that of the salivary gland ACCs. *KDM6A* mutations are often reported in salivary gland ACCs and facilitate novel gene-targeted therapy, including the use of BET and HDAC inhibitors.

**Conclusions:**

A better understanding of the underlying genetic mechanisms will help to clarify the carcinogenesis of BG-ACC. In turn, this will enable treatment with novel targeting agents, as well as the initial exploration of gene-based precision oncological therapies, which aim to improve treatment outcomes for patients with this disease.

## Background

Bartholin gland carcinomas (BGCs) are extremely rare tumors, comprising less than 1% of total malignancies in female genitals [1]. They have been categorized histologically as adenocarcinomas and squamous, adenosquamous, and adenoid cystic carcinomas (ACCs) [1]. Among these, adenocarcinomas and squamous carcinomas are the most common, accounting for approximately 40% of cases, whereas adenosquamous and ACCs comprise 5 and 15%, respectively [1]. BGCs have no distinguishing clinical manifestations; therefore, they are difficult to suspect or diagnose during clinical practice. Consequently, most primary BGCs are misdiagnosed and erroneously treated as benign Bartholin gland cysts or abscesses. Even with early correct diagnosis and successful local management, most patients die because of metastases within 10 years [1]. BGCs often metastasize to neighboring nerves, a mechanism known as perineural invasion, which is indicative of poor prognosis and increases the chance of local recurrence [2, 3]. Patients with BGC have poor prognosis owing to a lack of standard treatments, including chemotherapy.

The pathogenesis and carcinogenesis of BGCs remains unclear. The histological classifications, including adenocarcinoma, squamous, and adenosquamous, are similar to those of cervical cancer. In previous studies, it has been shown that a proportion of the squamous type might be related to human papillomavirus (HPV) infection, particularly HPV type 16, as well as to cervical cancer [2, 3]. However, due to a paucity of clinical trials, there is currently no consensus regarding the efficacy of radiation therapy, one of the approaches used to treat cervical cancer in patients with squamous-type BGC. Most ACCs occur in the salivary glands and it is an atypical histological type among genital cancers, although extremely rare cases have been reported in the breast, skin, respiratory system, and Bartholin glands [4]. Currently, given the rarity of this disease, the lack of prospective trials, and unknown carcinogenesis, valid chemotherapies do not exist for the long-term management for patients with BGCs.

Precision medicine, such as that using comprehensive genome sequencing, is a possible treatment approach for a number of cancers. In particular, recent molecular and whole-exome sequencing is beginning to reveal the genetic basis of this uncommon disease [5–8].

To date, genetic profiling for BG-ACCs has not been reported. In the present study, we analyzed 160 cancer-related genes from two patients with BG-ACCs as an attempt to bridge the current knowledge gap regarding the genetic characteristics of this disease. This information will help to clarify the carcinogenesis of BG-ACCs and will be useful to identify potential therapeutic targets for future precision medicine.

## Case presentation

Patient 1 was a 63-year-old woman undergoing excision of a vulvar lesion based on the diagnosis of a Bartholin gland cyst. The resected specimens were reviewed by a pathologist, who diagnosed it as a stage I adenoid cystic carcinoma in the Bartholin glands (BG-ACC). The patient was not provided with adjuvant therapy because there were no residual tumor tissues. However, metastasis to the lung was observed 4 years later. Although the patient had undergone video-assisted thoracoscopic surgery, recurrence at a site in the vulva was detected 5 years after this surgery. She subsequently underwent excision of the recurrent vulvar lesion, which was used for molecular analysis.

Patient 2 was a 61-year-old woman undergoing biopsy for a vulvar lesion and was diagnosed as having stage IV BG-ACC. She subsequently underwent total pelvic exenteration with resection of the inferior ramus. The patient had no adjuvant therapy because there were no residual tumors. However, metastases to the lung and liver were observed 2 years later. A biopsy obtained from the recurrent lesion in the liver was used for molecular analysis to establish an appropriate treatment approach.

**Fig. 1:**
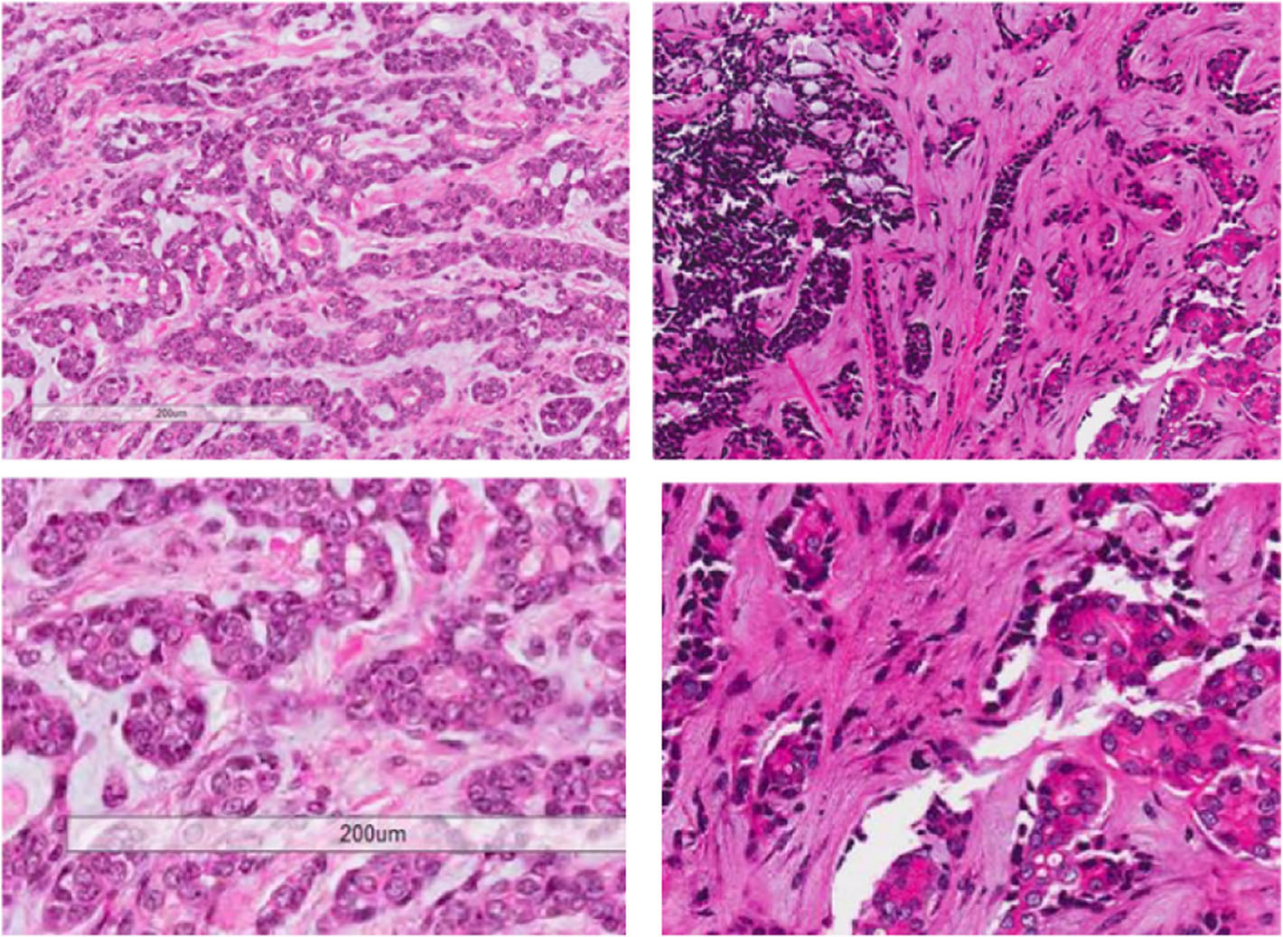
Histology of Bartholin gland adenoid cystic carcinomas. Cells were found to be organized in nests, sheets, and trabeculae, with a cribriform pattern (low-power view; case 1: upper left, case 2: upper right). We also observed comparatively homogeneous cells in nests and sheets with a cribriform pattern containing unstructured eosinophilic basement membrane-like components (high-power view; case 1: lower left, case 2: lower right)

Similar pathological findings were obtained for the tumor in case patient 1 and one of the tumors in the case patient 2. Low-power views showed tumor cells organized in nests, sheets, and trabeculae, with a cribriform pattern (Fig. 1; case 1: upper left, case 2: upper right). Higher power views showed nests and sheets of relatively uniform cells with a cribriform pattern. The cribriform regions comprised unstructured eosinophilic basement membrane-like components (Fig. 1; case 1: lower left, case 2: lower right). Expression of p16 was not detected in either tumor. Based on a pathologist’s assessment of the resected samples, the masses were determined to be BG-ACCs (case 1: pT1NxM0, case 2: pT1NxM0).

Genomic DNA sequences were obtained for the two samples classified as BG-ACCs, with average sequencing depths of 706.5× and 695.3× for patient 1 and patient 2, respectively. The average tumor cellularity was 30 and 80%, respectively, as determined histologically. Tumor cellularities estimated using a variant allele frequencies were 30 (case and 80% as well. Profiles of the gene alterations detected in each sample are shown in Table 1.

**Table 1:** Gene alterations in tumor samples

Case	Gene alteration	VAF (%)	CN	Pathogenicity
1	*KRAS* G12D	12.4	2.20	Pathogenic
2	*KDM6A c.2832 + 1G > C*	41.9	2.06	Pathogenic

*VAF* Variant allele frequency, *CN* Copy number.

Actionable gene alterations were detected in each sample. A *KRAS* point mutation was detected in the tumor from patient 1, and a *KDM6A* splicing alteration was detected in the tumor from patient 2 (Table 1). Details of these variants are described in Supplemental Figs. S1 and S2. No gene amplification or loss was detected in either sample. Of these two variants, the *KDM6A* mutation is potentially druggable. For both samples, the tumor mutation burden calculated from our pipeline was 1.3 single-nucleotide variants per megabase. Copy-number variation box and variant allele frequency plots are provided in Supplementary Figs. S3 and S4. With respect to the secondary germline, no ACMG-recommended genes for testing were observed in either patient.

## Discussion and conclusions

We sequenced 160 cancer-related genes in tumor samples obtained from two patients diagnosed with BG-ACC, and accordingly, detected *KRAS* and *KDM6A* mutations. Given that p16 expression was detected in neither of the samples, we hypothesize that HPV infection, which has been associated with squamous cell carcinomas [1, 9], was probably not involved in carcinogenesis in either of the two patients.

In these cases, we focused on the association between histological types and genetic events. The histological type of both cases was ACC, an uncommon malignancy that can arise in more than one organ site, despite being observed most often in the salivary glands [4]. For salivary gland ACCs, a few alterations have been identified in known cancer-related genes implicated in chromatin regulation, Notch signaling, and a number of other pathways, including *PIK3CA*, *ATM*, and *TSC1* [7, 10, 11]. Furthermore, recent studies have demonstrated a recurrent t(6;9)(q22–23;p23–24) translocation arising from the fusion of the v-myb myeloblastosis viral oncogene homolog (*MYB*) gene located on chromosome 6 with the nuclear factor I/B (*NFIB*) gene located on chromosome 9 in 44% of ACC cases [5, 11, 12]. However, given that analyses of the *MYB/NFIB* fusion gene have been performed for ACC mainly in the salivary gland, it remains unclear whether this gene is associated with the carcinogenesis of BG-ACC. The degree of contribution to the disease by other genes, and thereby the utility of the genes as possible therapeutic targets, is uncertain. Further, the extent to which other genes contribute to this disease and might constitute additional targets for potential therapeutic exploitation has not been well established, as previous genetic investigations have focused on salivary gland ACCs [10], and no similar sequencing has been performed with respect to BG-ACC.

In the report of Stephens et al., multiple mutations were identified in half of the examined cases, which collectively implicated chromatin deregulation [7]. In addition, somatic gene mutations were discerned in previously identified cancer-associated genes, including *PIK3CA*, *ATM*, *CDKN2A*, *SF3B1*, *SUFU*, *TSC1*, and *CYLD*. Togashi et al. detected *MYB* or *MYBL1* locus rearrangements in nearly all ACCs examined, suggesting that these might constitute good diagnostic markers for ACCs [12]. However, these authors found that the fusion transcript-specific RT-PCR for *MYB-NFIB* and *MYBL1-NFIB* and ordinary split FISH assays for *MYB* and *MYBL1* were less sensitive [12]. In our BG-ACC patient 1, we identified a *KRAS* mutation, which, to the best of our knowledge, has not been previously detected in salivary gland ACCs; this suggests differences between the carcinogenesis of BG-ACC and salivary gland ACCs [7, 11].

In our BG-ACC patient 2, we identified a mutation in *KDM6A*, which is related to chromatin remodeling. Previous studies have found alterations in genes implicated in histone modification and chromatin remodeling in cancer development [13]. Studies have reported somatic mutations in *ARID1A* in clear-cell, renal, transitional-cell bladder, and gastric carcinomas [13–16], as well as mutations in *CREBBP* and *EP300* in non-Hodgkin lymphoma [17] and in *KDM6A* in renal and transitional cell bladder carcinomas [13, 14]. However, to date, only truncating mutations of these genes have been reported. These findings, indicating that chromatin regulators are consistently altered in ACC, suggest an important role for the epigenetic control of gene expression in ACC and that the genetic profile of BG-ACC could be similar to ACCs of the salivary gland [6, 7]. Furthermore, it has been previously demonstrated that the loss of *KDM6A* triggers super-enhancers that cause sex-specific squamous-like pancreatic cancer with sensitivity to bromodomain and extra-terminal motif (BET) protein inhibitors [18]. Because histone pathology has a marked role in this pathway, novel compounds that target histone-related biomarkers might merit further study. For example, romidepsin, a histone deacetylase (HDAC) inhibitor, is an approved therapy for cutaneous and peripheral T cell lymphomas [19] with potential applications for ACC therapy. Given that a number of other pathways have also been linked to ACC development, grouping patients by molecular and histologic subtype will be key to increase our understanding of the clinical, pathological, and molecular correlations among them. However, it is not known whether these shared pathways are indicative of the potential effectiveness of combining current therapies. If this turns out to be the case, fundamental downstream pathways linked to proliferation, cell cycle regulation, angiogenesis, and cell adhesion also suggest possible therapeutic routes. Alternatively, the use of mutations with a lower prevalence in ACC than in other cancers (e.g. *SMARCA2*) as drug targets for BG-ACC might require further investigation and confirmation. Moreover, there is increasing evidence from human cancer genome sequencing that *KDM6A* participates in oncogenesis, suggesting it as a therapeutic target for BG-ACCs.

The recent and growing interest in the unique genetic pathways involved in neoplasia is partially a consequence of the promising developments in targeted therapies, which contribute to the increasing potential of personalized-medicine strategies. Previous studies have discovered a number of genetic abnormalities associated with oncogenes and tumor suppressor genes in the tumorigenesis of multiple cancer types. Given that no alterations in the RAS/ERK signaling pathway, which includes *KRAS,* have been detected in salivary gland ACCs to date, the detection of a *KRAS* mutation in the tumor of one of our patients suggests that we might have discovered a novel pathway related to BG-ACC carcinogenesis, which could in turn provide a novel therapeutic target.

Our study is limited by both its retrospective nature, which lacked information on fusion genes including *MYB/NFIB*, as well as by the limited number of patients with BG-ACC. Nonetheless, this is the first study reporting the genomic profiling of BG-ACC. Further genetic analyses of BGCs will undoubtedly elucidate the carcinogenesis of BGC and BG-ACC and will thereby provide a basis for novel gene-targeted therapy as a step toward developing precision medicine strategies.

## Supplementary information

**Additional file 1: Figure S1.** Details of the KRAS point mutation. **Figure S2.** Details of the KDM6A alteration. **Figure S3.** (Copy-number alteration and variant allele frequency (VAF) in case 1. The horizontal axis corresponds to each examined gene and the vertical axis corresponds to (A) copy number or (B) VAF. **Figure S4.** Copy-number alteration and variant allele frequency (VAF) in case 2. The horizontal axis corresponds to each examined gene and the vertical axis corresponds to the (A) copy number or (B) VAF.

**Additional file 2: Table S1.** Genes (160) examined in the PleSSision test

## Data Availability

The datasets used and analyzed during the current study are available from the corresponding author on reasonable request.

## Abbreviations

BCG: Bartholin gland carcinoma
ACC: Adenoid cystic carcinoma
HPV: Human papilloma virus
BET: Bromodomain and extra-terminal motif
HDAC: Histone deacetylase
