# Supplementary material for: Genetic profiling of patients with adenoid cystic carcinoma of the Bartholin’s glands reveals potential new routes for targeted therapies: a case report
Source: Diagn Pathol. 2020 May 28;15:64. doi: 10.1186/s13000-020-00976-2 (PMC7257184; doi:10.1186/s13000-020-00976-2)
Supplement: Supplementary file 1 — Additional file 1: Figure S1. Details of the KRAS point mutation. Figure S2. Details of the KDM6A alteration. Figure S3. (Copy-number alteration and variant allele frequency (VAF) in case 1. The horizontal axis corresponds to each examined gene and the vertical axis corresponds to (A) copy number or (B) VAF. Figure S4. Copy-number alteration and variant allele frequency (VAF) in case 2. The horizontal axis corresponds to each examined gene and the vertical axis corresponds to the (A) copy number or (B) VAF. [file 13000_2020_976_MOESM1_ESM.pptx]

## Slide 1
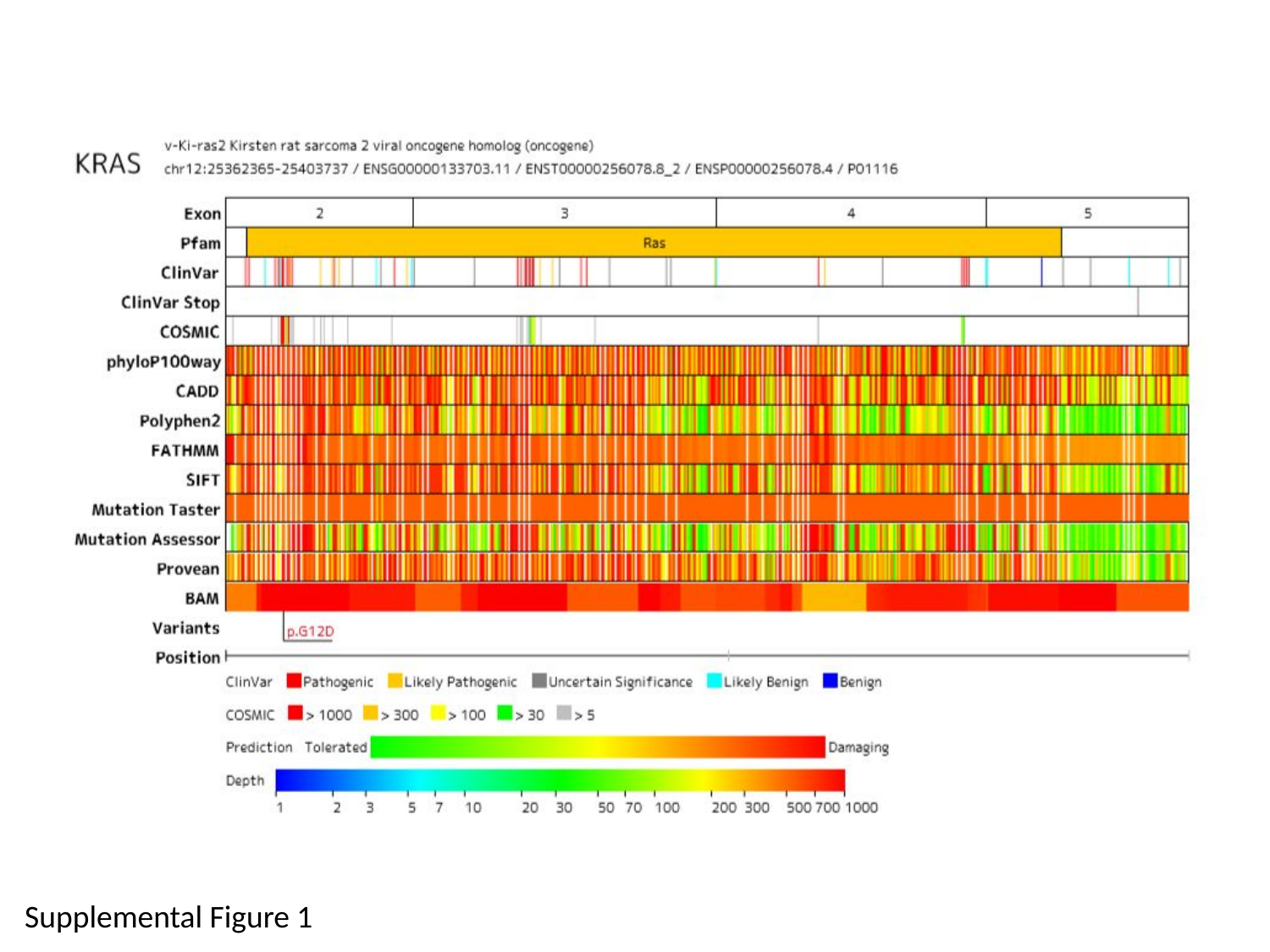

Supplemental Figure 1

## Slide 2
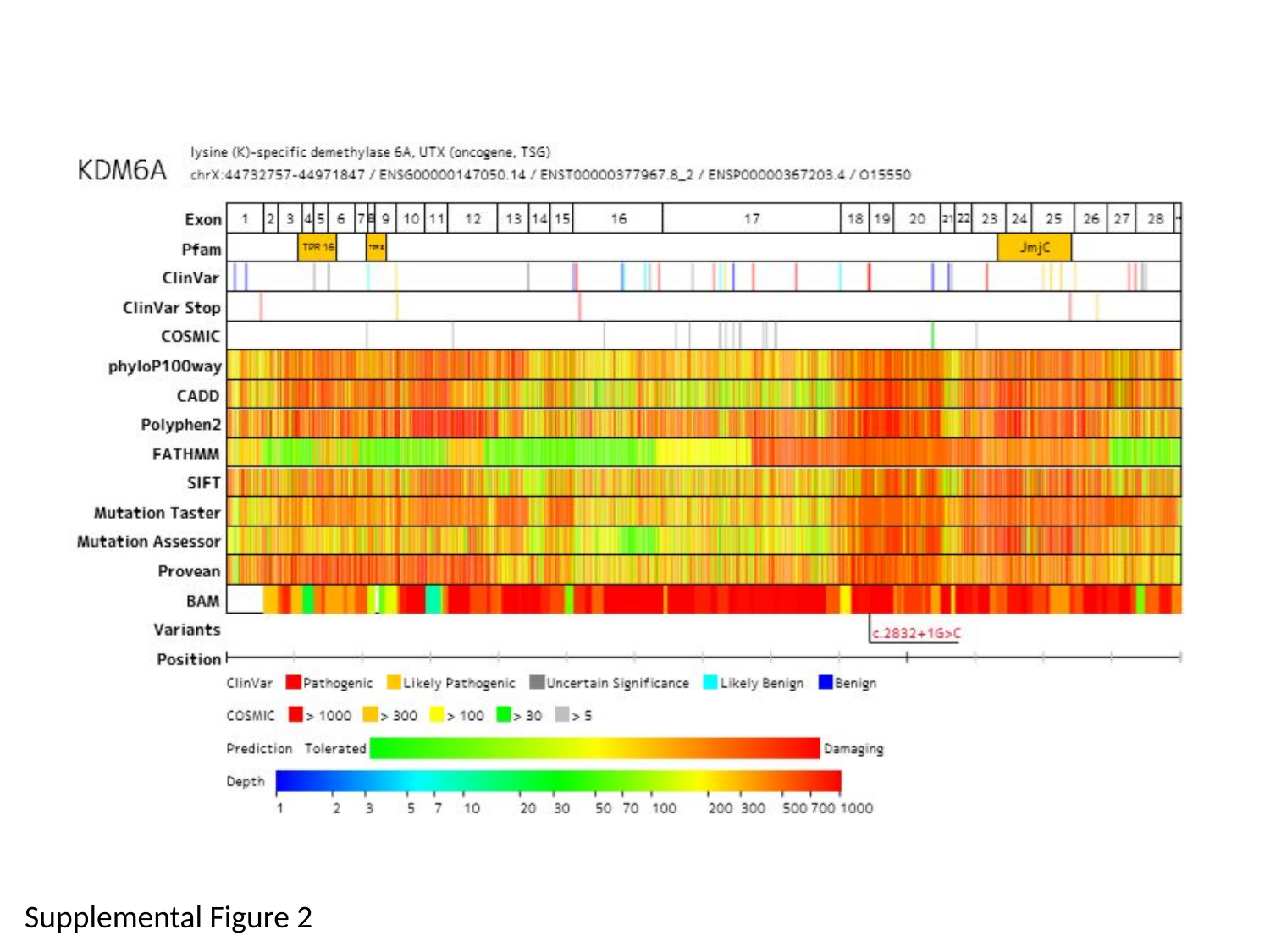

Supplemental Figure 2

## Slide 3
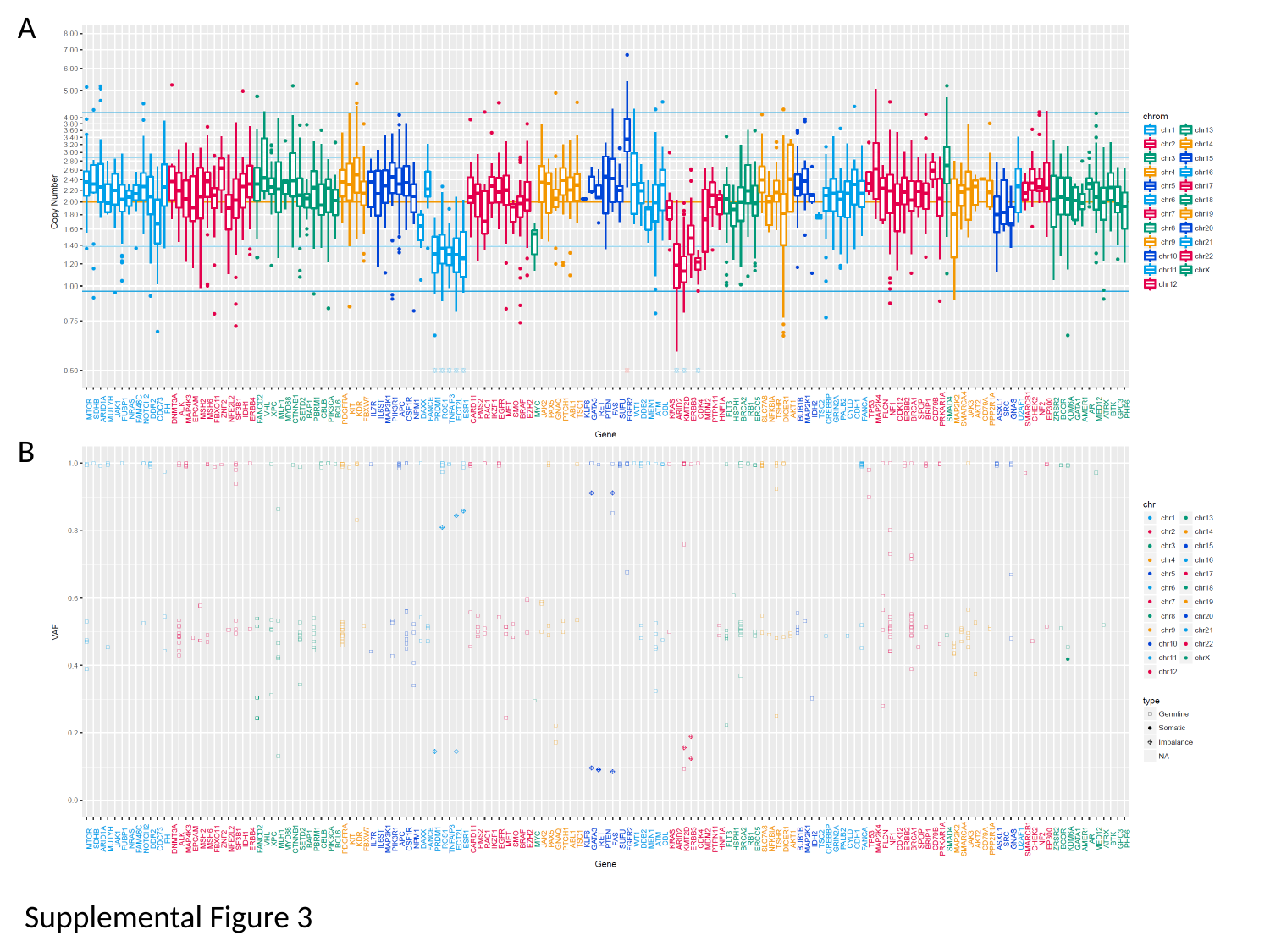

A
B
Supplemental Figure 3

## Slide 4
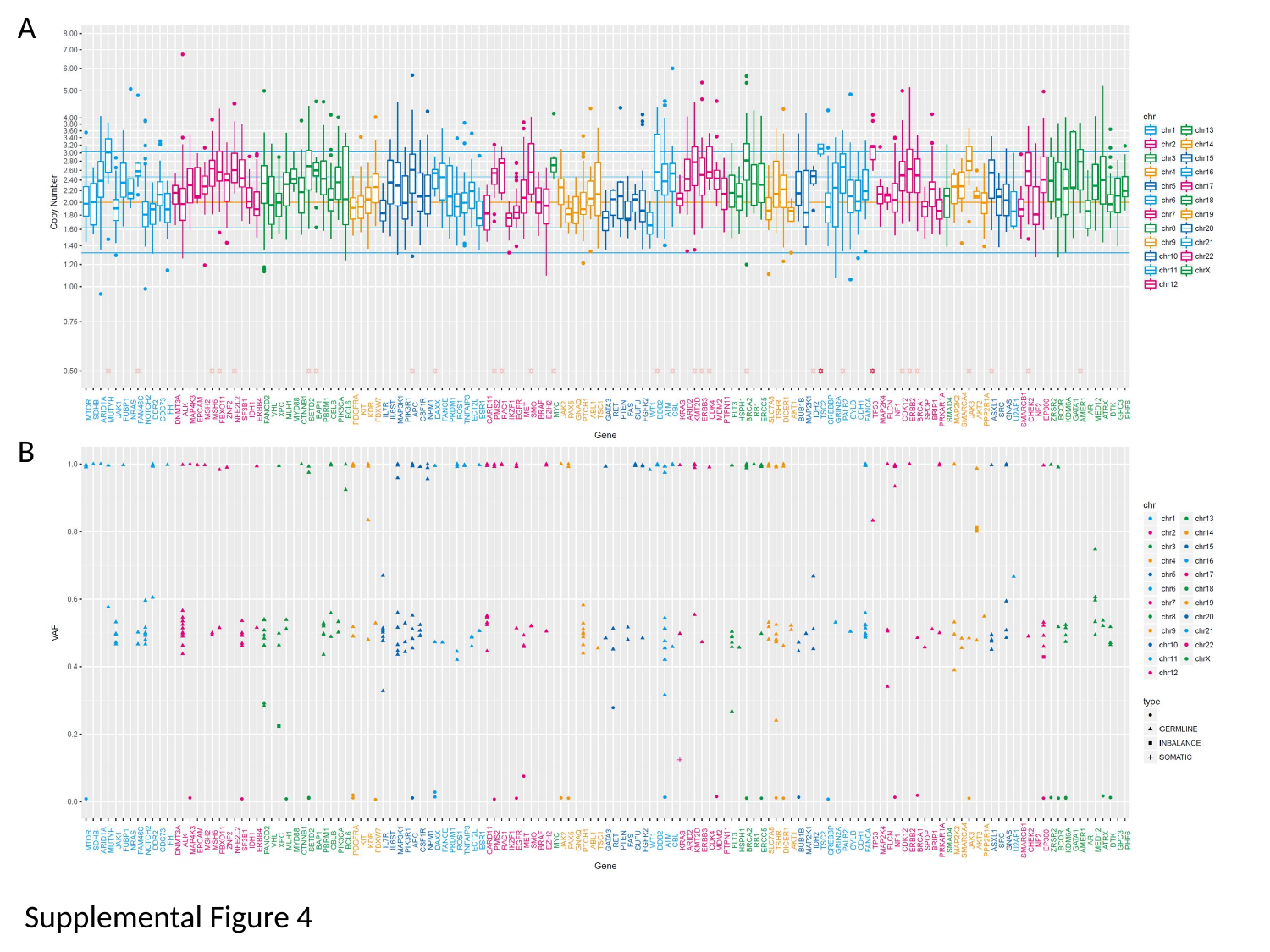

A
B
Supplemental Figure 4
