# Supplementary material for: Genetic profiling of patients with adenoid cystic carcinoma of the Bartholin’s glands reveals potential new routes for targeted therapies: a case report
Source: Diagn Pathol. 2020 May 28;15:64. doi: 10.1186/s13000-020-00976-2 (PMC7257184; doi:10.1186/s13000-020-00976-2)
Supplement: Supplementary file 2 — Additional file 2: Table S1. Genes (160) examined in the PleSSision test [file 13000_2020_976_MOESM2_ESM.zip › Supplementary_material_ver.2__without_track_change_.docx]

Supplementary material

Genetic profiling of patients with Bartholin’s gland carcinoma reveals potential new routes for targeted therapies: A case report

Kohei Nakamura et al.

We investigated the occurrence of mutations in 160 cancer-related genes in the tumor. Sections (10 µm) were dissected to provide > 20% tumor cells in the specimens and minimize the presence of necrosis. Genomic testing was performed on a PleSSision internal clinical sequencing apparatus (Keio University, Tokyo, Japan), which is used for all genome sequencing-related analyses in Keio University Hospital. This apparatus was used to extract genomic DNA from tumor samples and peripheral blood mononuclear cells extracted from cancer patients, following the provision of consent to receive comprehensive genomic testing. This study was conducted in accordance with the Declaration of Helsinki and Title 45, U.S. Code of Federal Regulations, Part 46, Protection of Human Subjects, effective December 13, 2001.

DNA quality was checked by calculating the DNA integrity number (DIN) using an Agilent 2000 TapeStation (Agilent Technologies, Waldbronn, Germany) prior to conducting targeted amplicon exome sequencing of the 160 genes using the Illumina MiSeq sequencing platform (Illumina, San Diego, CA). The genes examined are listed in Supplementary Table S1. The smallest quantity of DNA had a DIN greater than 3.1. Sequencing data were entered into the GenomeJack bioinformatics pipeline (Mitsubishi Space Software, Tokyo, Japan) for analysis. Cancer-specific changes in somatic genes, including SNVs, insertions/deletions, and copy number variations were detected and used to determine the TMB. In our system, secondary germline findings could be identified by comparing the genomic profiles obtained for tumor tissues and peripheral blood mononuclear cells.

Supplementary Figure legends

Figure S1: Details of the *KRAS* point mutation.

Figure S2: Details of the *KDM6A* alteration.

Figure S3: (Copy-number alteration and variant allele frequency (VAF) in case 1. The horizontal axis corresponds to each examined gene and the vertical axis corresponds to (A) copy number or (B) VAF.

Figure S4: Copy-number alteration and variant allele frequency (VAF) in case 2. The horizontal axis corresponds to each examined gene and the vertical axis corresponds to the (A) copy number or (B) VAF.

Supplementary Table legends

Table S1: Genes (160) examined in the PleSSision test
