# Supplementary material for: Genetic profiling of patients with adenoid cystic carcinoma of the Bartholin’s glands reveals potential new routes for targeted therapies: a case report
Source: Diagn Pathol. 2020 May 28;15:64. doi: 10.1186/s13000-020-00976-2 (PMC7257184; doi:10.1186/s13000-020-00976-2)
Supplement: Supplementary file 2 — Additional file 2: Table S1. Genes (160) examined in the PleSSision test [file 13000_2020_976_MOESM2_ESM.zip › Supplementary_Table_S1.docx]

**TABLE S1.** Genes examined in the PleSSision test

| ABL1 | AKT1 | AKT2 | ALK | AMER1 | APC | AR | ARID1A |
| --- | --- | --- | --- | --- | --- | --- | --- |
| ARID2 | ASXL1 | ATM | ATRX | BAP1 | BCL6 | BCOR | BRAF |
| BRCA1 | BRCA2 | BRIP1 | BTK | BUB1B | CARD11 | CBL | CBLB |
| CD79A | CD79B | CDC73 | CDH1 | CDK12 | CDK4 | CDKN2A | CHEK2 |
| CIC | CREBBP | CRLF2 | CSF1R | CTNNB1 | CYLD | DAXX | DDB2 |
| DDR2 | DICER1 | DNMT3A | ECT2L | EGFR | EP300 | EPCAM | ERBB2 |
| ERBB3 | ERBB4 | ERCC5 | ESR1 | EZH2 | FAM46C | FANCA | FANCD2 |
| FANCE | FAS | FBXO11 | FBXW7 | FGFR2 | FGFR3 | FH | FLCN |
| FLT3 | FUBP1 | GATA1 | GATA2 | GATA3 | GNA11 | GNAQ | GNAS |
| GPC3 | GRIN2A | H3F3A | HIST1H3B | HNF1A | HRAS | HSPH1 | IDH1 |
| IDH2 | IKZF1 | IL6ST | IL7R | JAK1 | JAK2 | JAK3 | KDM6A |
| KDR | KIT | KLF6 | KMT2D | KRAS | MAP2K1 | MAP2K2 | MAP2K4 |
| MAP3K1 | MAP4K3 | MDM2 | MED12 | MEN1 | MET | MLH1 | MSH2 |
| MSH6 | MTOR | MUTYH | MYC | MYD88 | NF1 | NF2 | NFE2L2 |
| NFKBIA | NOTCH1 | NOTCH2 | NPM1 | NRAS | PALB2 | PAX5 | PBRM1 |
| PDGFRA | PHF6 | PIK3CA | PIK3R1 | PMS2 | PPP2R1A | PRDM1 | PRKAR1A |
| PTCH1 | PTEN | PTPN11 | RAC1 | RB1 | RET | ROS1 | SDHB |
| SETD2 | SF3B1 | SLC7A8 | SMAD4 | SMARCA4 | SMARCB1 | SMO | SPOP |
| SRC | STK11 | SUFU | TERT | TNFAIP3 | TNFRSF14 | TP53 | TSC1 |
| TSC2 | TSHR | U2AF1 | VHL | WT1 | XPC | ZNF2 | ZRSR2 |
